# (Fluoro)quinolone prescriptions for upper respiratory tract infections in the German outpatient sector: a health insurance claims analysis

**DOI:** 10.3205/id000091

**Published:** 2025-05-27

**Authors:** Birgit Arens, Helmut L’hoest, Alissa Wolf, Beata Hennig, Ursula Marschall, Irit Nachtigall

**Affiliations:** 1 Kompetenz-Centrum Qualitätssicherung, Medizinischer Dienst Baden-Württemberg, Lahr, Germany; 2 Barmer Institut für Gesundheitssystemforschung, Medizin und Versorgungsforschung, Berlin, Germany; 3 Vivantes Netzwerk für Gesundheit GmbH, Head of Translationale Forschung, Lehre und Kooperation, Berlin, Germany

**Keywords:** upper respiratory tract infections, fluoroquinolones, side effects, general practices, health insurance data, Germany

## Abstract

**Background:**

Acute upper respiratory tract infections (URTIs) are frequent causes of medical treatment by general practitioners (GPs). In general, these are viral and self-limiting illnesses, but antibiotics are prescribed. In Germany, fluoroquinolones are not authorised for the indications pharyngitis, laryngitis, tonsillitis and acute bronchitis due to possible serious side effects. This analysis looks at fluoroquinolone prescriptions for URTI by GPs in 2022.

**Methods:**

Frequency of fluoroquinolone prescriptions were analysed at case level and regionally using administrative insurance data from the German health insurance company BARMER. We included patients treated in GP practices without relevant concomitant diseases.

**Results:**

In 2022, 25% (1,197,568/4,720,786) of insured persons with upper respiratory tract infections (URTI) were prescribed antibiotics. After excluding comorbidities and other risk factors such as previous hospitalisation, the prescription rate for uncomplicated URTIs was calculated to be 6% (80,786/1,365,646). Fluoroquinolones were given in 2.4% of antibiotic prescriptions (1,951/80,786). Nationwide, 3.7% of the GPs prescribed fluoroquinolones, most frequently in the region Brandenburg with 6.6% (74/1,121).

**Conclusion:**

This analysis showed that fluoroquinolones are still prescribed for uncomplicated URTI, which must be considered as alarming regarding the nature of the disease and the unfavourable risk-benefit profiles. There were clear regional differences in fluoroquinolone prescribing, indicating potential for improvement in the use of reserve antibiotics for uncomplicated upper respiratory tract infections.

## Background

Quinolones were discovered in the early 1960s and the addition of a fluorine atom to the quinolone core in the 1970s led to the development of the first fluoroquinolone flumequine and further chemical modifications to the 2^nd^ generation quinolones such as ciprofloxacin and norfloxacin. In the meantime, the 4^th^ generation of quinolones has been developed, including moxifloxacin and delafloxacin [1]. There are different proposals how to define the quinolone generations. Besides the classification by clinical use and spectrum of antibacterial activity by Bush et al. [1] the Paul-Ehrlich-Society (PEG) classified fluoroquinolones based on the spectrum of antimicrobial activity, pharmacokinetics and clinical indications into four groups [2]. Delafloxacin, an anionic fluoroquinolone effective against MRSA and not yet available on the German market, shows promising properties that discussions of establishing a 5^th^ group are emerging. Newer fluoroquinolones are broad-spectrum antibiotics that are effective against various gram-positive and gram-negative bacteria. The spectrum of action of the individual fluoroquinolones differs. Fluoroquinolones are associated with various side effects of the central nervous system, the heart, the gastrointestinal tract and tendons, muscles and joints. Side effects such as aortic aneurysms, liver toxicity, imbalance of glucose metabolism or Clostridium difficile infections have also been reported [3]. Side effects can occur within 48 hours but also after a delay of several months following fluoroquinolone administration. In the past, fluoroquinolones such as sparfloxacin, temafloxacin or trovafloxcin have been withdrawn from the market due to side effects [4]. Five fluoroquinolones developed for systemic use (norfloxacin, ofloxacin, ciprofloxacin, levofloxacin and moxifloxacin) are currently available on the German market [5].

The World Health Organisation (WHO) has listed fluoroquinolones as critically important antibiotics [1]. As the first choice antibiotic in German outpatient care fluoroquinolone can be considered only for a few special indications, e.g. pyelonephritis in younger men if the resistance to *Escherichia coli* is below 10%, prostatitis, moderate community-acquired pneumonia or severe chronic obstructive pulmonary disease (COPD) and special reasons such as beta-lactam allergy [6], [7], [8]. According to a study by the AOK Research Institute (WidO), around 5% of all statutorily insured persons in Germany received at least one fluoroquinolone therapy in 2017 and 2018. This leads to the assumption that fluoroquinolone antibiotics are not used as a reserve drug or exclusively for serious and life-threatening infections [5]. Due to increasing safety concerns, the risk-benefit profile of fluoroquinolone was reassessed by the European Medicines Agency (EMA) between 2017 and 2018. In 2019, the EMA banned the marketing of some quinolones such as nalidixic acid and restricted the use of systemic and inhaled fluoroquinolone antibiotics [9]. Fluoroquinolone should not be used for infections that may resolve without treatment at all or are not serious, such as sore throat, non-bacterial infections, to prevent traveller’s diarrhoea or recurrent lower urinary tract infections or to treat mild or moderate bacterial infections, unless other common antibiotics cannot be used. These restrictions on prescribing have been implemented by individual European countries. In Germany, healthcare professionals have been informed several times by red-handed letters [10], [11]. Fluoroquinolones are no longer authorised in EU member states (including Germany) for diseases such as pharyngitis, laryngitis, tonsillitis and acute bronchitis, and for acute bacterial rhinosinusitis only if the usually recommended antibiotics are unsuitable [12].

## Objective

Using administrative insurance data from a statutory health insurance company in Germany, we analysed how frequently fluoroquinolones were prescribed for acute uncomplicated upper respiratory tract infections in 2022 and whether the results can be used to derive quality improvement potentials in the outpatient sector. The data was analysed at the level of patient cases, general practitioners (GPs) and federal states. 

## Material and methods

The analysed data is based on the anonymised administrative dataset of the Scientific Data Warehouse (W-DWH) of the BARMER Institute of Health System Research (bifg). The dataset included diagnostic data of general practitioners and hospitals for reimbursement purposes using the German Modification of the International Statistical Classification of Diseases (ICD-Code), 10^th^ Revision. The drug prescription data is encoded based on the Anatomical Therapeutic Chemical (ATC) codes. The ICD-10 diagnoses and prescriptions (ATC) for the years 2020 to 2022 for around 8.7 million BARMER insured persons in 2022 were used. Cases (≥20 years) with acute uncomplicated upper respiratory tract infections (URTIs) including acute bronchitis (index diseases), which were treated in the outpatient sector by GPs in 2022 were analysed (as described in [13]).

### Population: inclusion- and exclusion criteria

The following URTI diagnoses (index diseases; ICD-Codes) were considered:

- Acute nasopharyngitis (common cold) (J00)
- Acute sinusitis (J01)
- Acute pharyngitis (J02.8 and J02.9)
- Acute tonsillitis (J03.8 and J03.9)
- Acute laryngitis and tracheitis (J04)
- Acute upper respiratory infections (URI) of multiple and unspecified sites (J06)
- Acute bronchitis (J20.3 to J20.9)

Only statutorily insured persons who were continuously insured in the previous 2 years before 2022 and could be assigned to an Associations of Statutory Health Insurance Physicians (ASHIP) region were considered.

In order to identify only acute and uncomplicated URTI cases, all insured persons who met one of the following criteria were excluded from the analyses (Supplementary Table S1 in Attachment 1):

- Index quarter:
  - Several index diseases
- Index and previous quarter:
  - In-hospital treatment
  - Acute comorbidity
- Index quarter or previous two years:
  - Chronic comorbidity

Cases include outpatients of GPs. GPs in Germany are typically self-employed and work in their own practices. In 2022 there were around 55,112 GPs registered by the National Associations of Statutory Health Insurance Physicians, of which 29,249 GPs (53%) were working in a solo ambulatory practice, 21,136 GPs (38%) in a dual practice and 4,665 GPs (9%) in a multispecialty medical care centre [14], [15]. By our data analysis, 49,750 GPs (90%, range by region 80% to 97%) were included, which were working in 30,127 practices. GPs were identified by the German lifetime doctor number and had to match the medical speciality of the practice. Only medical practices that treated ≥three index cases and had a proportion of ≥50% GPs in the practice were included in the calculation at outpatient practice level. Doctor’s practices including laboratory medicine and/or microbiology specialists were excluded (as described in [13]). The aim of the comprehensive exclusion algorithm was to generate a healthy population with nearly no comorbidities which therefore is not representative of a GP’s population. The proportion of practices where GPs described a fluoroquinolone for acute URTI was investigated, as fluoroquinolones should not be prescribed for acute uncomplicated URTIs in otherwise healthy persons. 

Only systemic fluoroquinolones (ATC J01MA) were considered. The first antibiotic prescription for the index disease that was prescribed by the URTI-diagnosing practice was analysed. In order to determine the percentage of cases with antibiotic prescriptions, the first prescriptions and index cases were analysed after adjustment for all excluded cases.

### Descriptive statistics

The results presented here are descriptive and exploratory. Antibiotic prescription rates are reported as the ratio of cases with antibiotic prescription to all cases in the same diagnostic group in 2022. These categorical characteristics are given as frequency and percent.

Risk adjustment was carried out in order to generate a sample of a highly specific population with most uncomplicated URTI infections possible. The broad spectrum of exclusion diagnoses for potentially risky disease courses and factors beyond the practitioner’s control (Supplementary Table S1 in Attachment 1) were used to maximise specificity (as described in [13]) .

### Extrapolation to the German population

The dataset used only covers part of the statutory health insurance population in Germany. The BARMER population of insured persons does not fully correspond to the insured German population in terms of age and gender distribution and regional structure. In order to extrapolate to the insured German population, the reimbursement and prescription data was weighted by age and gender standardisation to the statutory health insurance population. The KM6 statistics (German Membership Statistics Statutory Health Insurance) were used as a basis for this.

### Presentation at regional level

In Germany, outpatient treatment is reimbursed by the local ASHIP. The ASHIP in general corresponds to the federal states, besides North Rhine-Westphalia, were two ASHIPs, North Rhine and Westphalia-Lippe, exist. In the areas of the federal states Bremen, Hamburg and Saarland the number of patients insured by the BARMER health insurance company is low. This can lead to a distortion at the federal state level due to small numbers. Therefore, these three regions were not analysed at this level. 

## Results

**Figure 1:**
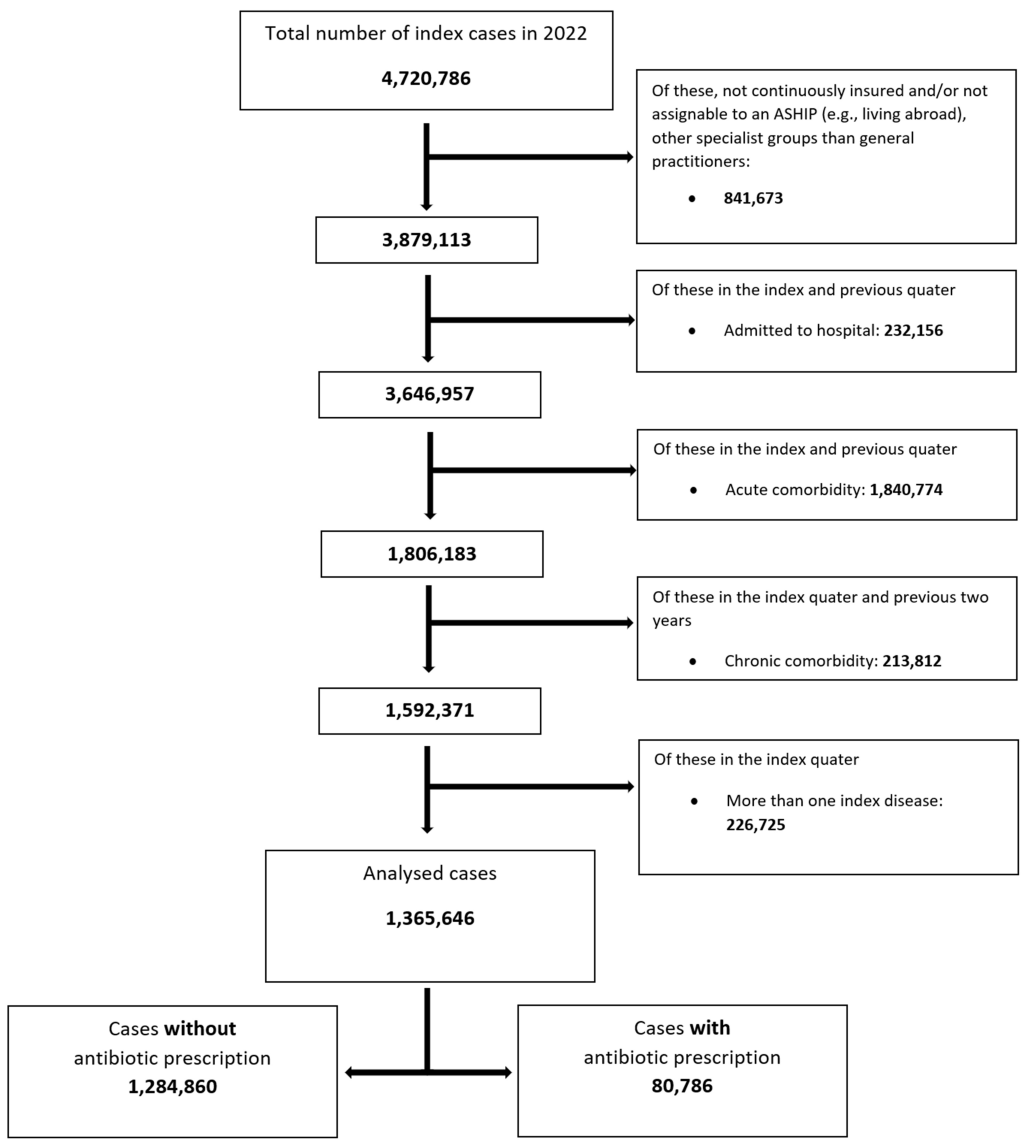
Flowchart of analysed cases

Figure 1 (Fig. 1) illustrates the inclusion and exclusion process. The aim of this process was to identify acute uncomplicated URTIs that do most likely not require antibiotic therapy. The algorithm leads to a significant reduction in the evaluable acute URTI cases from 4,720,786 to 1,365,646. The antibiotic prescription rate for acute uncomplicated URTIs fell from 25% (1,197,568/4,720,786) in the unfiltered population to 6% (80,786/1,365,646) after algorithm application. Fluoroquinolones were prescribed in 1.08% (50,908/4,720,786) of the unfiltered population and decreased to 0.14% (1,951/1,365,646) in the filtered population. The algorithm comprehensively excludes comorbidities that could justify any antibiotic prescription in individual cases.

Acute upper respiratory infections (URI) of multiple and unspecified sites (J06) were with 85.7% (1,169,965/1,365,646) one of the most frequently coded diagnoses of acute uncomplicated URTI, followed by common cold (J00) with 6.2% (84,243/1,365,646) and acute bronchitis (J20.3 to J20.9) with 2.8% (38,913/1,365,646).

### Fluoroquinolone prescriptions at case level

**Figure 2:**
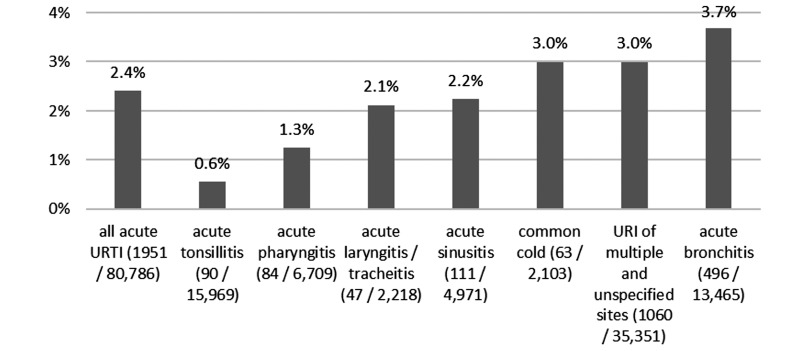
Fluoroquinolone share of all prescribed antibiotics, displayed by diagnosis (2022)

Among the 80,786 cases with antibiotic prescriptions, fluoroquinolones were prescribed in 1,951 cases across all diagnoses, which corresponds to a proportion of 2.4%. Fluoroquinolones were prescribed most frequently for acute bronchitis with a share of 3.7%, and least frequently for acute tonsillitis with a share of 0.6% (Figure 2 (Fig. 2)).

Fluoroquinolones commercially available in Germany are ciprofloxacin, levofloxacin, moxifloxacin, ofloxacin and norfloxacin. With a share of 50.1% (977/1,951) ciprofloxacin was the most frequently prescribed fluoroquinolone, followed by levofloxacin with a share of 31.1% (607/1,951) and moxifloxacin with a share of 16.9% (330/1,951).

### Fluoroquinolone prescriptions at general practitioner level

In total 30,127 practices of GPs could be considered in the analysis. Practices that had less than three cases per index diagnosis were not included in the analysis. This leads to a reduction in both the number of cases and of fluoroquinolone prescriptions at practice level. 

**Figure 3:**
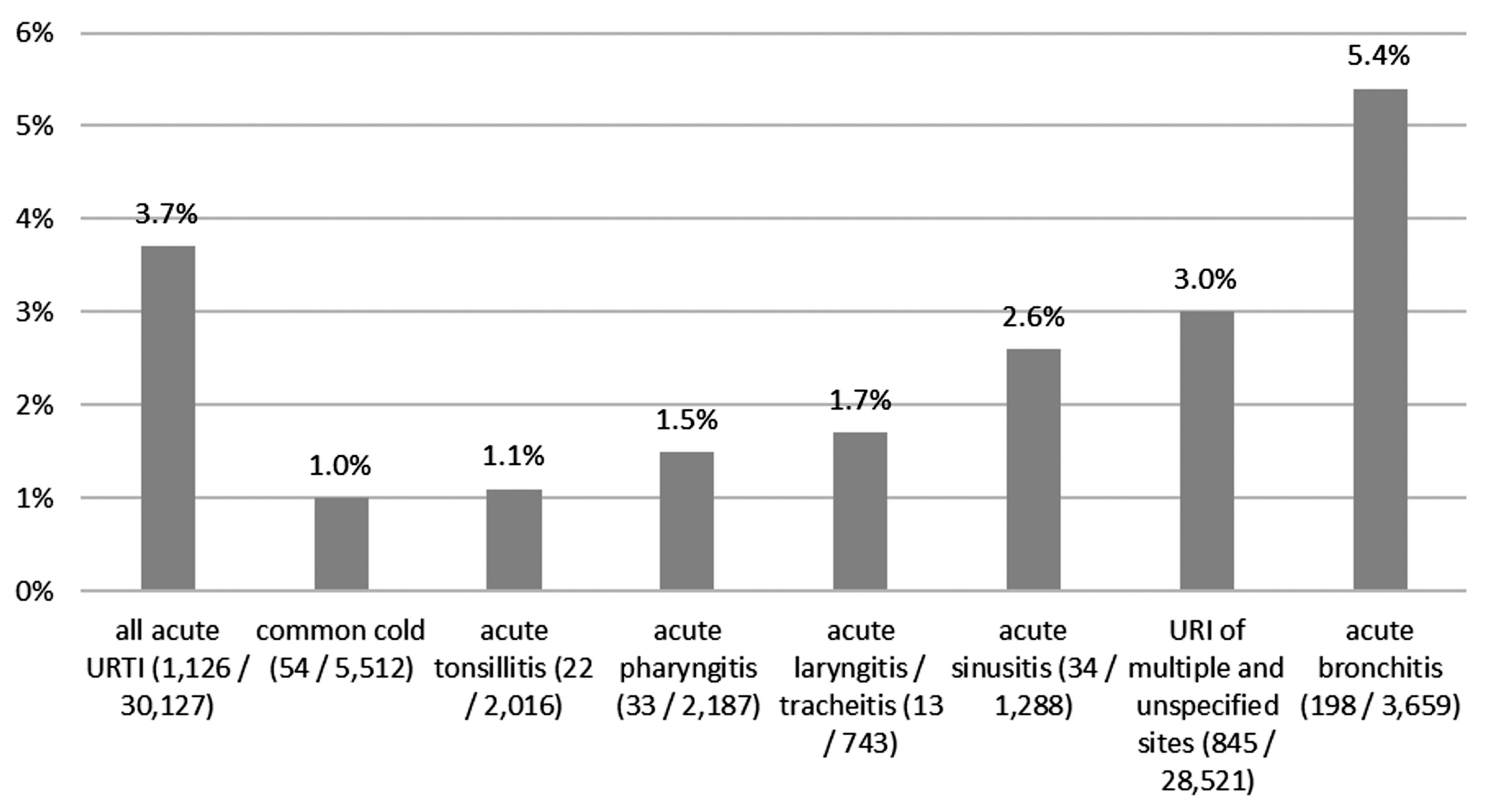
Share of practices prescribing fluoroquinolone among all general practitioners’ practices, displayed by diagnosis (2022)

Across all index diseases, 3.7% (1,126/30,127) of all included practices in Germany prescribed fluoroquinolones for acute upper respiratory tract infections. Fluoroquinolones were prescribed most frequently by 5.4% (198/3,659) of practices for acute bronchitis and by 3.0% (845/28,521) of practices for URI of multiple and unspecified sites (Figure 3 (Fig. 3)). 

**Figure 4:**
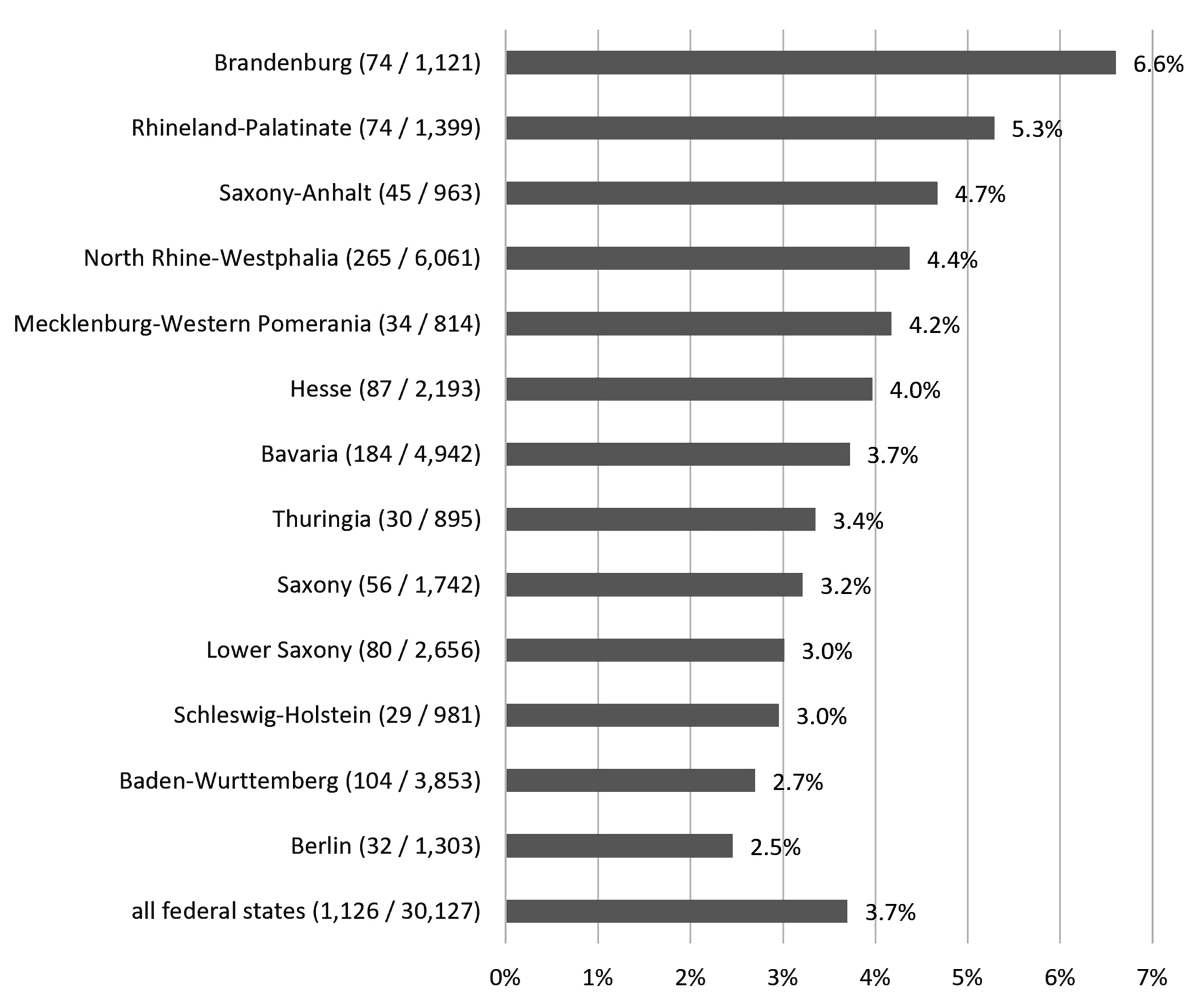
Proportion of general practices prescribing fluoroquinolone among all general practices, displayed by federal states (2022). The federal states of Bremen, Hamburg and Saarland are not shown due to the low number of people insured by the BARMER health insurance company in these regions.

At the regional level, 6.6% (74/1,121) of practices in Brandenburg and 5.3% (74/1,399) of practices in Rhineland-Palatinate prescribed a fluoroquinolone. The proportion of prescribing practices in these two federal states is the highest compared to the others and is well above the national average of 3.7%. No value is given for the federal states of Bremen, Hamburg and Saarland due to the low number of people insured by the BARMER health insurance company in these regions. Figure 4 (Fig. 4) shows the share of practices prescribing fluoroquinolones for index diseases among all practices displayed by federal states. 

### Data extrapolation for German population

The 1,951 cases with fluoroquinolone prescriptions in 2022 are distributed over 1,773 patients. In 2022, the BARMER health insurance company had a share of around 12% of the statutorily insured people aged 20 years and older which is around 10% of the total German population. The age- and gender-stratified extrapolation of the 1,773 patients to all statutorily insured adults aged ≥20 years results in an estimate of 14,927 patients who received a fluoroquinolone prescription for uncomplicated upper respiratory tract infections in 2022.

**Figure 5:**
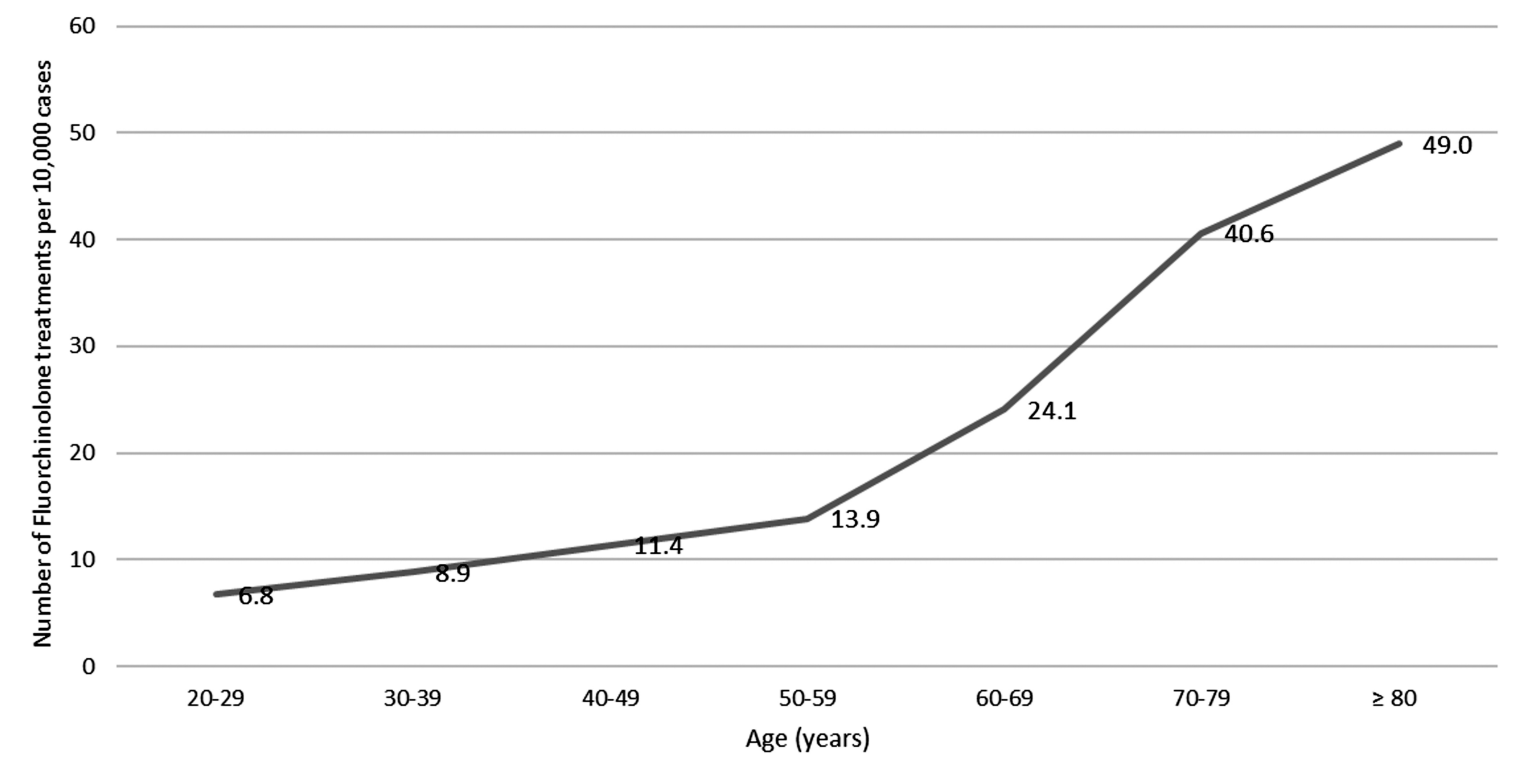
Extrapolated number of fluoroquinolone treatments per 10,000 cases with acute uncomplicated upper respiratory tract infection displayed by age group (2022)

Of the extrapolated 14,927 patients nationwide, 47.4% (7,074) were male and 52.6% (7,854) female, which corresponds very closely to the gender distribution of the statutorily insured German population aged ≥20 years (47.5%/52.5%). Looking at the age groups, there is an increase in fluoroquinolone prescriptions with age. In the age group 60 to 69 years old, there is an increase in prescriptions to 24.1 prescriptions per 10,000 treatment cases. The highest number of fluoroquinolone prescriptions is found in patients ≥80 years with 49 prescriptions per 10,000 acute upper respiratory tract infections (Figure 5 (Fig. 5)).

## Discussion

Acute respiratory infections were common reasons for antibiotic prescriptions in the outpatient sector until 2018 [16]. Following a risk assessment for systemic fluoroquinolones by the EMA, the use of fluoroquinolones for the indications pharyngitis, laryngitis, tonsillitis and acute bronchitis was revoked in 2019 [12], [17]. 

In our analysis, serious underlying diseases and comorbidities were comprehensively and specifically excluded. Therefore cases analysed were predominantly uncomplicated acute URTI in otherwise healthy individuals. The prescription of antibiotics is generally not recommended for these patients [18], [19], [20]. In this analysis, 1,773 BARMER insured persons with uncomplicated acute URTI in 2022 received systemic fluoroquinolones. The proportion of GP practices prescribing fluoroquinolones varies regionally from 2.5% to over 6% of all included GP practices.

The ESAC-NET group developed quality indicators concerning antibiotic prescription for acute uncomplicated respiratory tract infections [21]. One of these quality indicators regards the proportion of quinolones in all prescribed antibiotics for URTIs and sets the quality target at a share of <5% quinolones among all prescribed antibiotics. In 2019, fluoroquinolones accounted for 6.2% of all prescribed antibiotics across all indications in the outpatient sector in Germany [22].

In our analysis, fluoroquinolones accounted for 4.25% of all antibiotic prescriptions (50,908/1,197,568) in the unfiltered population in 2022, which is below the target range of 5% of the ESAC-NET quality indicator. However, the target range of the ESAC-NET indicator dates back to 2011 [21] and might no longer be appropriate for uncomplicated acute URTIs following the EMA’s reassessment of serious adverse events in 2019. In our analyses, we included acute bronchitis, which is a lower tract infection. There exists a smooth transition between a common cold and an acute bronchitis and both have mainly a viral cause. However, in 5 to 10% bacterial agents like *Bordetella pertussis*, *Mycoplasma pneumoniae* and *Chlamydia* *pneumoniae* can cause an acute bronchitis [23]. For these bacterial pathogens, fluoroquinolones or macrolides might be useful. In our analysed population, ciprofloxacin was prescribed most frequently. However, ciprofloxacin has its spectrum of activity predominantly in the gram-negative range, including Pseudomonas aeruginosa. Therefore, it should not be prescribed for acute URTIs without risk factors for a Pseudomonas aeruginosa infection. 

In 2023, the German Federal Institute for Drugs and Medical Devices (Bundesinstitut für Arzneimittel und Medizinprodukte, BfArM) issued a red-handed letter reminding of restrictions on fluoroquinolone use and stating that current study data indicate that fluoroquinolones continue to be prescribed outside the recommended indications [11]. This assumption appears to be confirmed by the data of our analysis.

The AOK Research Institute estimates that, compared to other antibiotics, an additional 1,161 side effects of the nervous system (mainly confusion and restlessness), 33 tendon ruptures, 8.2 aortic aneurysms and 4 cardiovascular deaths may occur among 100,000 fluoroquinolone users [5]. If these numbers of assumed side effects are applied to the 14,927 patients with acute upper respiratory tract infections in our extrapolation for Germany, the following potential number of additional complications in 2022 might be statistically expected: 173.3 side effects of the nervous system, 4.9 tendon ruptures, 1.2 aortic aneurysms and 0.6 cardiovascular deaths. Even if these figures initially appear low, it should be borne in mind that these are theoretically avoidable side effects in a presumably healthy population, as extensive comorbidities were excluded through the algorithm used.

Our data showed clear regional differences in the prescription of fluoroquinolones. Fluoroquinolones were most frequently prescribed for acute uncomplicated URTIs in the more rural regions Brandenburg and Rhineland-Palatinate. Regional differences for antibiotic prescriptions were also found in other analyses, with more frequent prescriptions of cephalosporins and quinolones in the new German states [22], [24]. Kern assumed that around 15% of regional differences in antibiotic prescribing for URTIs cannot be explained solely by differences in age and morbidity [25]. 

## Conclusion

The regional differences in fluoroquinolone prescriptions indicate that there is further potential for optimising the prescription of reserve antibiotics. The use of fluoroquinolones for acute uncomplicated URTIs should be avoided whenever possible due to the potentially severe side effects, the partial lack of authorisation and their status as reserve antibiotics. 

## Limitations

The use of routinely collected reimbursement data has limitations. The primary purpose of coding is reimbursement between providers and health insurance companies. Accordingly, in addition to the professional medical information, the common coding practice is mapped. Statements on care quality can be distorted by incentives in reimbursement [13].

Diagnosis codes are often not specific enough to adequately differentiate between pre-existing and concomitant conditions. We therefore comprehensively excluded possible comorbidities and concomitant conditions, which could justify an antibiotic prescription and could not be influenced by healthcare providers. The analysed collective is therefore unlikely to represent a typical GP population [13].

2022 was characterised by the highest number of Covid-19 cases to date [26]. In 731,875 cases of our analysed population, Covid-19 was also coded in the index quarter. A preliminary analysis showed that the proportion of antibiotic prescriptions for the population with Covid-19 infection was slightly lower than for the population without Covid-19 infection. Since Covid-19 is a viral infection and showed predominantly mild courses in outpatient treatment in 2022, these cases were not excluded. We did not test for group differences [13].

In this analysis, a penicillin allergy could not be validly operationalised. Several codes are possible in German ICD-10. It is assumed that a penicillin allergy would not have a significant influence on fluoroquinolone prescriptions. Besides fluoroquinolones, other antibiotics of different classes were available if an antibiotic prescription would have been necessary [13].

## Notes

### Supplementary information

The table with the inclusion and exclusion criteria according to [13] is available in Attachment 1.

### Authors’ contributions

B.A.: conceptualization, drafting of the manuscript, H.L.: formal analysis, data curation, validation, A.W.: conceptualization, visualization, validation, B.H.: data curation, validation, U.M.: data curation, validation, I.N.: conceptualization, validation.

All authors commented on previous versions of the manuscript. All authors reviewed, edited and approved the final manuscript.

### Data availability

The insurance data is protected by the German data protection law. Uncensored data cannot be made available. The database is not publicly accessible.

### Ethics approval and informed consent

This research was conducted retrospectively and used only fully anonymized data from the German BARMER health insurance company. Therefore, no ethics approval was required (as per German law) and no patient consent had to be obtained. 

### Competing interests

The authors declare that they have no competing interests.

The Center of Excellence for Quality Assurance (Kompetenz-Centrum Qualitätssicherung, KCQ) advises the National Association of Statutory Health Insurance Funds (GKV-Spitzenverband, GKV-SV) on questions of healthcare quality strategies at the Federal Joint Committee (Gemeinsamer Bundesausschuss, G-BA).

## Supplementary Material

Supplementary Table S1
