# Supplementary material for: (Fluoro)quinolone prescriptions for upper respiratory tract infections in the German outpatient sector: a health insurance claims analysis
Source: GMS Infect Dis. 2025 May 27;13:Doc01. doi: 10.3205/id000091 (PMC12171980; doi:10.3205/id000091)
Supplement: Supplementary Table S1 [file ID-13-01-s-001.pdf]

## Attachment 1

**Supplementary Table S1: Inclusion and exclusion criteria according to [13]**

| Inclusion criteria | Exclusion criteria |
|---|---|
| Index diseases:<br>• Acute nasopharyngitis [common cold] (J00)<br>• Acute sinusitis (J01)<br>• Acute pharyngitis (J02.8 and J02.9)<br>• Acute tonsillitis (J03.8 and J03.9)<br>• Acute laryngitis and tracheitis (J04)<br>• Acute upper respiratory infections of multiple and unspecified sites (J06)<br>• Acute bronchitis (J20.3 to J20.9)<br><br>Physicians:<br>• Family doctors (general medicine, general internists, general practitioners)<br>• Lifelong physician number matches the practice specialty<br>• URTI diagnosing practice prescribed 1st antibiotic in the quarter of the index disease | • Not continuously insured at BARMER in the past two years and/or cannot be assigned to any Associations of Statutory Health Insurance Physicians region (Kassenärztliche Vereinigung) (e.g. living abroad)<br><br>In the index and previous quarter:<br>• Hospital admission<br>• Acute comorbidities:<br>➢ Various infections A00 to A99<br>➢ Cystitis N30, Other disorders of urinary system N39.0, Disorder of urinary system, unspecified N39.9<br>➢ Nonsuppurative otitis media H65<br>➢ Suppurative and unspecified otitis media H66<br>➢ Osteomyelitis M86<br>➢ Inflammatory diseases of prostate N41<br>➢ Streptococcal tonsillitis J03.0<br>➢ Streptococcal pharyngitis J02.0<br>➢ Influenza and pneumonia J09–J18<br>➢ Acute bronchitis due to Mycoplasma pneumonia J20.0<br>➢ Acute bronchitis due to Haemophilus influenzae J20.1<br>➢ Acute bronchitis due to streptococcus J20.2<br>➢ Acute bronchiolitis J21<br>➢ Unspecified acute lower respiratory infection J22<br>➢ Other diseases of the upper respiratory tract J30–39<br>➢ Suppurative and necrotic conditions of lower respiratory tract J85–J86<br>➢ Other diseases of pleura J90–J94<br>➢ Other diseases of the respiratory system J95–J99<br>➢ Pulmonary embolism I26<br>➢ Human immunodeficiency virus [HIV] disease B20–B24<br>➢ Diseases of the blood and blood-forming organs and certain disorders involving the immune mechanism D50–D70<br>➢ Diabetes with difficulties E10–E14 with 0–8 in 4th place<br>➢ Pregnancy, childbirth and the puerperium O<br>➢ Care involving dialysis Z49<br>➢ Dysphagia R13 |

Attachment 1 to: *Arens B, L'hoest H, Wolf A, Hennig B, Marschall U, Nachtigall I. (Fluoro)quinolone prescriptions for upper respiratory tract infections in the German outpatient sector: a health insurance claims analysis. GMS Infect Dis. 2025;13:Doc01. DOI: 10.3205/id000091, URN: urn:nbn:de:0183-id0000912*

| Inclusion criteria | Exclusion criteria |
|---|---|
| | • Antibiotic prescription in the last month of the previous quarter before the index illness<br>In the index quarter and two previous years:<br>• Relevant chronic comorbidities:<br>➢ Malignant neoplasms C00–C97<br>➢ Certain disorders involving the immune mechanism D80–89<br>➢ Immune compromise due to radiation, chemotherapy or other immunosuppressive measures D90*<br>➢ Cystic fibrosis E84<br>➢ Chronic lower respiratory diseases J40–J47<br>➢ Acute tubulo-interstitial nephritis N10, Chronic tubulo-interstitial nephritis N11,<br>➢ Tubulo-interstitial nephritis, not specified as acute or chronic N12<br>➢ Senility R54<br>➢ On waiting list for organ transplant U55*<br>➢ Successful registration for organ transplantation without HU urgency status (High Urgency) Z75.6*<br>➢ Successful registration for organ transplantation with HU urgency status (High Urgency) Z75.7*<br>➢ Transplanted organ and tissue status Z94<br><br>• More than one of the index diseases under consideration in the quarter under consideration |
| **Additionally at the practice level** | |
| | • <3 index cases in 2022<br>• <50% general practitioners/practice<br>• Facilities with laboratory doctors, microbiologists |
| * Translated to German according to ICD-10-GM version 2022 with https://gesund.bund.de/en/icd-code-search | |

Attachment 1 to: *Arens B, L'hoest H, Wolf A, Hennig B, Marschall U, Nachtigall I. (Fluoro)quinolone prescriptions for upper respiratory tract infections in the German outpatient sector: a health insurance claims analysis. GMS Infect Dis. 2025;13:Doc01. DOI: 10.3205/id000091, URN: urn:nbn:de:0183-id0000912*
